# No side effects on rabbit retina or vitreous microenvironment by nd:YAG laser vitreolysis

**DOI:** 10.1186/s12886-024-03406-9

**Published:** 2024-04-16

**Authors:** Tiezhu Lin, Mingqin Zhang, Bing Wu, Yuanlong An, Emmanuel Eric Pazo, Rong Li, Lijun Shen

**Affiliations:** 1 Center for Rehabilitation Medicine, Department of Ophthalmology, Zhejiang Provincial People’s Hospital (Affiliated People’ Hospital), Hangzhou Medical College, Hangzhou, Zhejiang 310000 China; 2 He Eye Specialist Hospital, Shenyang, Liaoning Province China; 3 Jinzhou Medical University, Jinzhou, Liaoning Province China; 4 Dalian Medical University, Dalian, Liaoning Province China; 5 The School of Pharmacy, He University, Shenyang, Liaoning Province China; 6 Department of Ophthalmology, the First Affiliated Hospital, Xi’an Medical University, Xi’an, Shanxi Province China

**Keywords:** Floaters, Vision degrading myodesopsia, Nd:YAG laser vitreolysis, Vitreous microenvironment, Retinal pathology

## Abstract

**Background:**

To explore the safety of Neodymium:Yttrium-aluminum-garnet (Nd:YAG) laser vitreolysis based on the histological examination of the retina and the alteration of vitreous cytokines in the rabbits.

**Methods:**

Nine male New Zealand rabbits underwent Nd:YAG laser vitreolysis of 10 mJ x 500 pulses in the left eyes, while the right eyes were used as controls. Intraocular pressure, color fundus photography, and ultrasound B scan were measured before, as well as 1 day, 4 weeks, and 12 weeks after Nd:YAG laser vitreolysis. Three rabbits were euthanized 1 day, 4 weeks, and 12 weeks after treatment, respectively. Terminal deoxynucleotidyl transferase dUTP nick end labeling (TUNEL) staining and hematoxylin-eosin (H&E) staining were used to look for pathological changes in the retina. An enzyme-linked immunosorbent assay (ELISA) was utilized to detect the expression of vascular endothelial growth factor (VEGF) and some inflammatory cytokines, including interferon inducible protein 10 (IP-10), monocyte chemoattractant protein 1 (MCP-1) and interlenkin 6 (IL-6) in the vitreous humor. The ascorbic acid (AsA) and total reactive antioxidant potential (TRAP) in the vitreous humor were also measured.

**Results:**

Following Nd:YAG laser vitreolysis, the levels of VEGF, IP-10, MCP-1, IL6, AsA, and TRAP in the vitreous humor did not change substantially (*P* > 0.05). There were no detectable pathological changes in the retinal tissues, and no apoptotic signal was found.

**Conclusions:**

Rabbits tolerate Nd:YAG laser vitreolysis without observable impact on retinal tissue or the microenvironment of the vitreous.

## Introduction

The vitreous gel is a homogenous substance in infants that gradually degrades and condenses as individuals age or become more myopic. Subsequently, vitreous liquefaction, collagen fiber aggregation, and vitreous cortical separation from the retina occur. The fusing collagen fibers or a detached posterior vitreous membrane or Weiss ring could cause the bothersome vitreous floaters. This condition is extremely prevalent, with a reported prevalence of close to 76% [1]. Recently, the term vision degrading mydesopsia (VDM) has been frequently used to characterize this disease [2]. Despite the fact that the majority of patients with VDM are able to tolerate the symptoms of floaters [3], a subset of patients with impaired vision and quality of life seek therapy. Neodymium:Yttrium-aluminum-garnet (Nd:YAG) laser vitreolysis is an effective treatment for VDM [4–8].

On the other hand, the vitreous humor is a crucial ocular structure that plays a major role in sustaining biochemical homeostasis and consuming molecular oxygen [9]. Gel vitreous has a higher concentration of ascorbate (AsA) and utilizes oxygen at a faster rate than liquefied vitreous caused by myopia, aging, or surgical removal [10]. The vitreous humor contains high levels of antioxidants, specifically AsA and glutathione, according to previous research [9]. Due to the fact that the Nd:YAG laser treats vitreous floaters by vaporizing and shattering [11], some doctors worry that the vitreous body’s disruption by the Nd:YAG laser may alter the vitreous microenvironment, including VEGF, inflammatory factors, and ascorbic acid (AsA) concentrations, in addition to the retina [12]. In this study, a rabbit eye model of Nd:YAG laser vitreolysis was proposed to examine the safety of Nd:YAG laser vitreolysis by assessing changes in the levels of vitreous-related factors and retinal histopathology.

## Materials and methods

### Animals

**Fig. 1:**
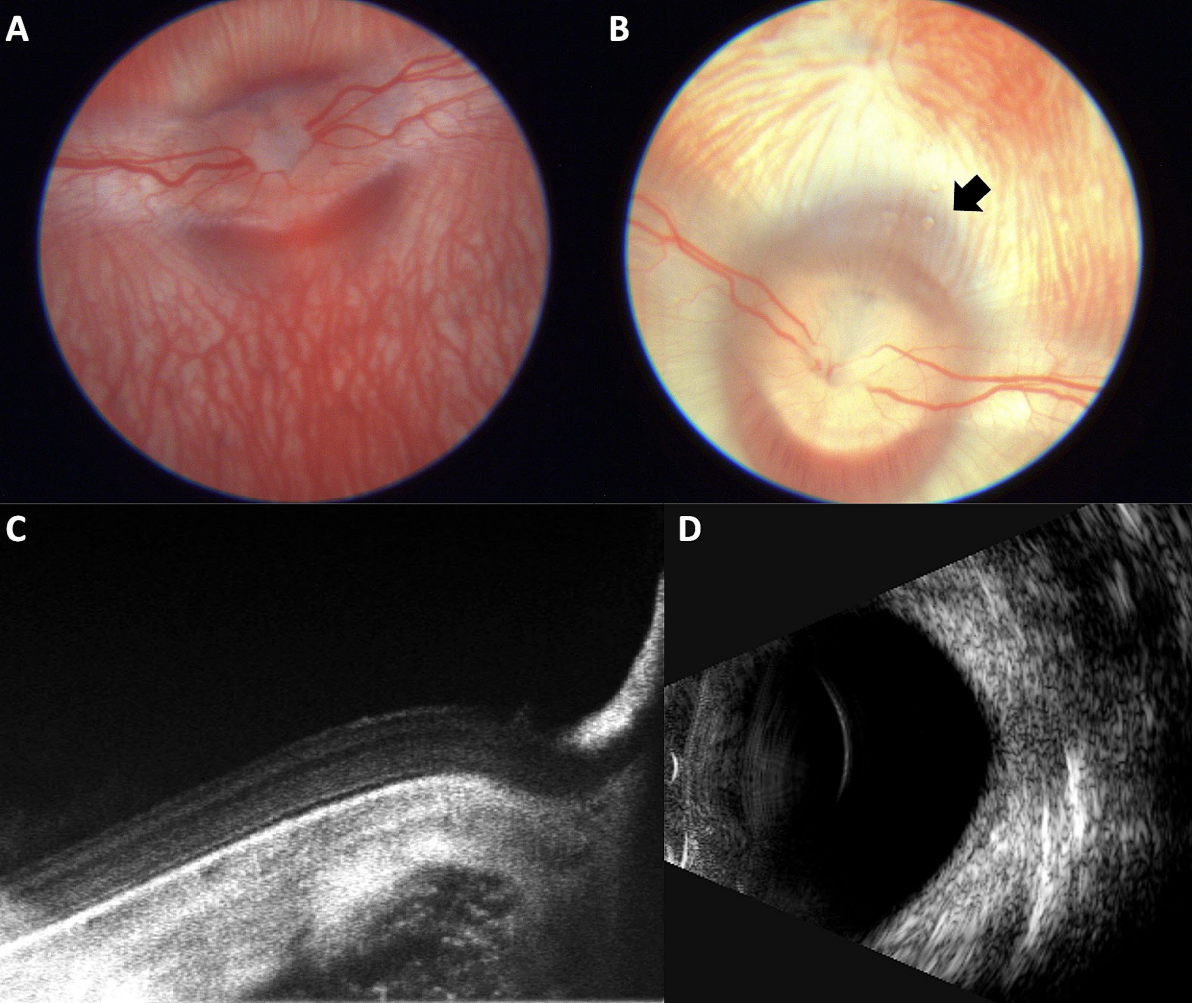
Retinal images following Nd:YAG laser vitreolysis. **A**. Color photo of the retina before Nd:YAG laser vitreolysis; **B**. Color photo of the retina after YAG laser vitreolysis, gas bubbles (black arrow) were noted above the optic disc; **C**. Retinal OCT of the treated eye three months after Nd:YAG laser vitreolysis, clear retinal layers were noted without any damage; **D**. Ultrasound B scan of the treated eye three months after Nd:YAG laser vitreolysis, no unmistakable echo of the vitreous cavity was found

In this investigation, nine adult male New Zealand rabbits (Liaoning Changsheng Biotechnology Co., LTD. ) were two months old and weighed between 2 and 3 kg [12–14]. All experiments were conducted in accordance with the Association for Research in Vision and Ophthalmology Statement for the Use of Animals in Ophthalmic and Vision Research and the guidelines of the institution. The rabbits were housed on a light/dark cycle of 12/12 hours, free of food and water, for 8 h before the start of the experiment. All rabbits received anesthesia via subcutaneous injection with a blend of xylazine hydrochloride and ketamine hydrochloride. The pupil was dilated using topical eye drops comprising 0.5% tropicamide and 0.5% phenylephrine hydrochloride. After induction of general and topical eye (0.4% hydrochloride) anesthesia, the rabbits underwent slit-lamp, intraocular pressure (IOP), color fundus photography (Fig. 1A), optical coherence tomography (OCT) and an ultrasound B scan before Nd:YAG laser vitreolysis and 1 day, 4 weeks, and 12 weeks after treatment to determine a potential change in IOP and transient effects on the vitreous and retina. Three rabbits were euthanized by injecting sodium pentobarbital intravenously (100 mg/kg) 1 day, 4 weeks, and 12 weeks after laser treatment, respectively. A 21-gauge needle was attached to a 2.0-ml tuberculin syringe and injected 2 mm posterior to the limbus into the mid-vitreous cavity. A sample of undiluted vitreous humor (0.5 ml) was manually aspirated and immediately transferred to a sterile tube, which was stored at − 80 °C until the assay was performed. Then the eyes were enucleated cautiously to avoid probable globe violations and stored in Davidson’s fixative solution for 24 h [15].

### Nd:YAG laser vitreolysis

Rabbits’ left eyes were treated with Nd:YAG laser vitreolysis, whereas the right eyes were used as controls. A Q-switched Nd:YAG laser (Ultra Q reflex, Ellex Medical Lasers, Adelaide, AU) was used in this experiment. The laser has a wavelength of 1064 nm and a pulse width of 4 ns. A mid-vitreous laser lens (Volk Singh Mid-Vitreous Lens, Volk Optical, US) was applied to the eye with gel (Ofloxacin Eye Ointment, Shenyang Sinqi Pharmaceutical, Shenyang, CN). The middle vitreous humor anterior to the visual streak area was focused with 15 degrees of oblique axis irradiation in reference to the thickness of the lens and the blurring retinal background. Each eye received a total of 500 spots with 10 mJ of pulse energy. Visible gas bubbles formed in the vitreous (Fig. 1B).

### Vitreous humor analyses

The concentrations of vascular endothelial growth factor (VEGF), interferon inducible protein 10 (IP-10), monocyte chemoattractant protein 1 (MCP-1) and interlenkin 6 (IL-6) were determined using a multiplex cytokine assay (Shanghai Enzyme-Linked Biotechnology, Shanghai, CN) per the manufacturer’s user guide. Each sample was measured three times, and an average was then determined.

AsA concentrations were determined using a colorimetric technique based on its decrease from Fe^3+^ to Fe^2+^ upon reaction with 2,2’-dipyridyl. Modifications to the assay allowed for the examination of 10 µl samples, and all measurements utilized a standard curve. As a control for AsA specificity, two units of ascorbate oxidase (Shanghai Enzyme-linked Biotechnology, Shanghai, CN) were added to extra 10 µl samples, thoroughly mixed at room temperature, and then AsA measurements were performed in triplicate.

Using the trolox equivalent antioxidant capacity (TEAC) method on a sample made in the same way as for thiobarbituric acid reactive substances (TBARS) measurements, the total reactive antioxidant potential (TRAP) of vitreous humor fed a diet high in verbascosides was found. Specifically, using the radical cation decolorization assay established by Re et al. [16], the total antioxidant activity of the samples was determined. A chemical reaction with potassium persulfate (K2S2O8) generated the 2,2’-azino-bis (3-ethylbenzothiazoline-6-sulfonic acid) (ABTS^+^) radical. The sample of 25 ml of ABTS (7 mM) was spiked with 440 ml of K2S2O8 (140 mM) and placed in the dark at room temperature for 12–16 h for the radicals to form. The concoction of the working solution was prepared by diluting a volume of the previously mentioned solution with ethanol until its absorbance at λ = 734 nm reached 0.70 ± 0.02.

The measurement was carried out with a Varian Cary 100UV-VIS spectrophotometer, and this instrument was then connected to a 25 °C thermostat basin. The reaction took place directly in the measuring cuvette. Here, 2 ml of the ABTS^+^ radical was added, the absorbance (A_0_) was measured, and promptly 100 µl of the sample or standard was added. The radical was inhibited by the antioxidants in the sample, resulting in a decrease in absorbance proportional to the antioxidant concentration in the sample. At each standard and sample dilution, triplicate measurements were performed, and the calculation for percentage inhibition was relative to the absorbance of the blank at 734 nm. The definition of total antioxidant activity of samples was the concentration of Trolox equating to mol/g sample.

### Hematoxylin–eosin (H&E) staining

**Fig. 2:**
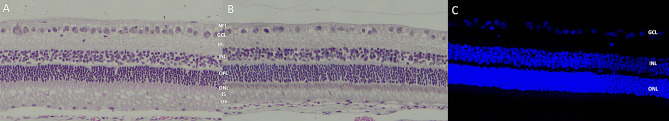
Retinal histological examination following Nd:YAG laser vitreolysis. **A**. H&E-stained retinal histological specimen of the control eye; **B**. H&E-stained retinal histological specimen of the treated eye; **C**. TUNEL-stained retinal histological specimen of the treated eye. NFL: Nerve Fiber Layer; GCL: Ganglion Cells Layer: IPL, Inner Plexiform Layer: INL, Inner Nuclear Layer: OPL, Outer Plexiform Layer: ONL: Outer Nuclear Layer; IS: Inner Segment and OS: Outer Segment

After fixation, the eyes were cut in half along the anterior-posterior axis. Following the completion of histological processing, H&E was used to stain the 4 μm-thick specimens, which were viewed with the help of a light microscope (Fig. 2A). A minimum of three retinal tissue sections were evaluated, with one of them exhibiting a visual streak [15]. The morphologies of the retinal pigment epithelium (RPE), photoreceptors, and ganglion cells were carefully examined. The same pathologist (Y.L.A.) examined each specimen.

### Terminal deoxynucleotidyl transferase dUTP nick end labeling (TUNEL) staining

In summary, after the retinal tissues were fixed in paraffin, slices of 4 μm thickness were stretched at 42 °C water to be set and baked. Next, the subsequent sections were added dropwise with the TdT reaction solution, and the reaction took place in the darkroom for one hour. Thenceforward, the reaction was stopped by incubation in deionized water for 15 min. Following the use of hydrogen peroxide to block the activity of endogenous peroxidase, the sections were added dropwise to the working solution. After the reaction was left for 1 h, the sections were washed, 3,3′-Diaminobenzidine (DAB) solution was added in drops for the color to develop, counterstained with hematoxylin for 20–30 s, and then washed again in running water. Finally, they were dehydrated at increasing alcohol concentrations and watched while sealed. The apoptotic index would be calculated if positive cells were noted.

### Statistical analysis

All statistical analyses were performed on SPSS version 27.0 (SPSS Inc., USA). The mean and standard error of the mean were used to present the data in this study. The Dunnett-t test was used to analyze the differences between the two groups, and one-way analysis of variance (ANOVA) was utilized to calculate multiple comparisons. *P* < 0.05 indicated statistical significance.

## Results

### Change of IOP

IOP measurements were compared to baseline at postoperative 1 day (12.67 ± 1.12 vs. 12.44 ± 1.24, *p* = 0.694), 4 weeks (12.67 ± 1.21 vs. 12.44 ± 1.24, *p* = 0.644), 12 weeks (12.33 ± 1.53 vs. 12.44 ± 1.24, *p* = 0.815), and no significant differences were found.

### Histology of retina with H&E and TUNEL staining

Normal thickness and morphology of ganglion cells, photoreceptors, RPE, and nuclear layers were observed in all specimens. There was no evidence of retinal edema or hemorrhage (Fig. 2B). TUNEL staining revealed no apoptosis in the retinal and choroid layers (Fig. 2C).

### Vitreous humor analysis

**Fig. 3:**
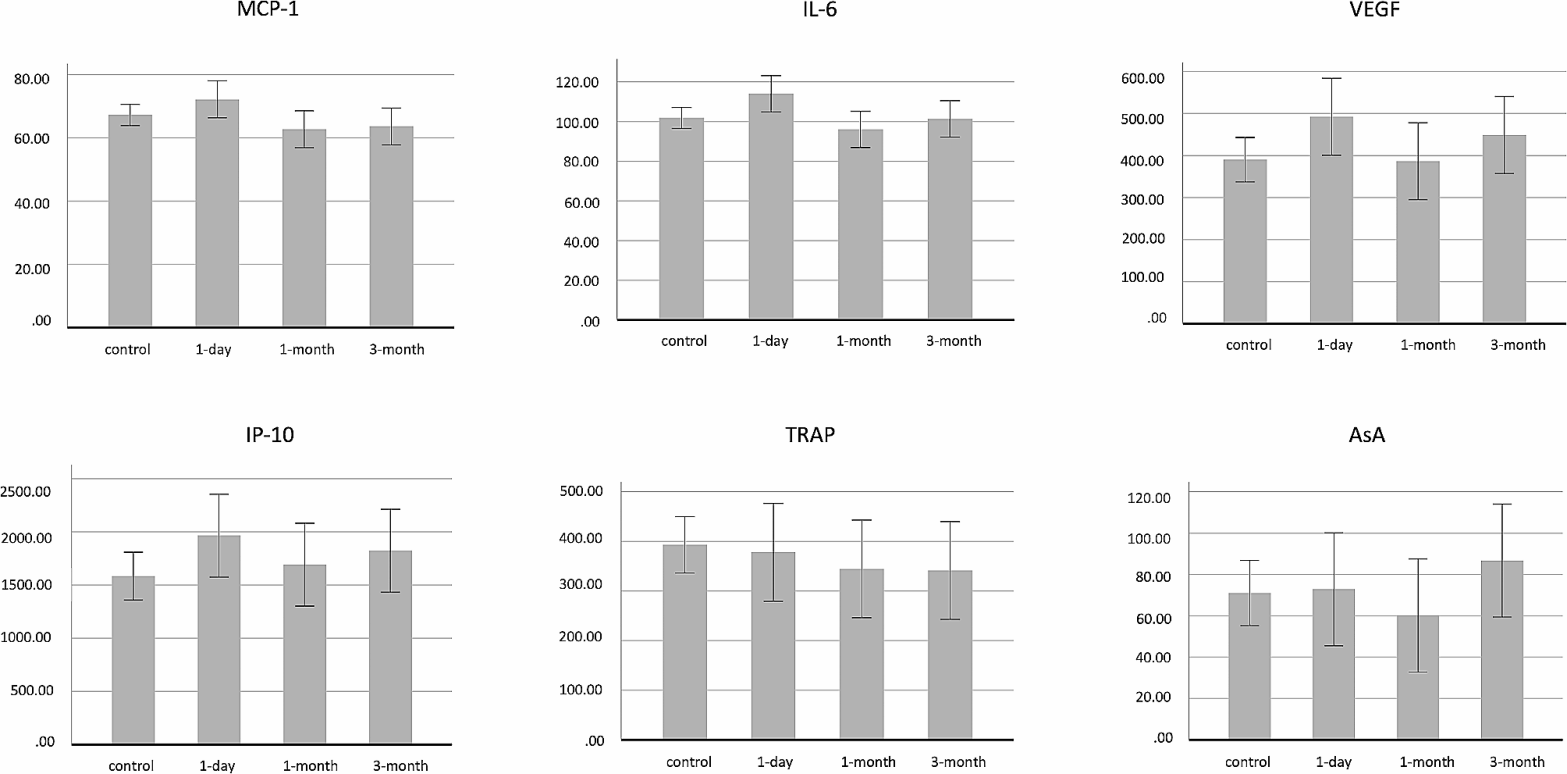
Vitreous levels of studied factors pre- and post-YAG laser vitreolysis, all *p* > 0.05. IP-10: Interferon-inducible protein 10, VEGF: Vascular endothelial growth factor, AsA: Ascorbic acid, TRAP: Total reactive antioxidant potential, MCP-1: Monocyte chemotactic protein‐1, IL‐6: Interleukin‐6

There were no differences in VEGF, IL-6, MCP-1, or IP-10 levels measured in vitreous humor specimens of collected eyes at 1 day, 4 weeks, or 12 weeks after Nd:YAG laser vitreolysis compared to control eyes (*p* > 0.05). There were no differences in either AsA or TRAP among all groups (*p* > 0.05) (Fig. 3).

### Other possible complications

Throughout the subsequent observation period, no external ocular inflammation, cataract development, or retinal abnormalities were detected in any of the eyes. OCT revealed that Nd:YAG laser vitreolysis did not cause any retinal injury (Fig. 1C). After Nd:YAG laser vitreolysis, the ultrasound B scan revealed no aberrant echo in the vitreous cavity (Fig. 1D).

## Discussion

In 1993, Tsai et al. [17] introduced Nd:YAG laser vitreolysis for treating floaters. Subsequently, Shah et al. [4] were the first to evaluate the safety and efficacy of this treatment in a randomized controlled trial. In their study, the symptomatic improvements of the treatment were found in 53% and 9% of patients in the Nd:YAG laser group and the control group, respectively. Moreover, the Nd:YAG laser group demonstrated a greater improvement in their 10-point visual disturbance score compared to the control group. Although the efficacy of Nd:YAG laser vitreolysis for VDM has been further demonstrated by some other prospective studies [5–8], several small case series and individual case reports have described some complications, such as transient elevation of intraocular pressure, refractory glaucoma, acute cataract formation, retinal detachment, retinal hemorrhage, and absolute scotoma [18]. Because the problems that can happen with Nd:YAG laser vitreolysis have only been described in case reports, it is not possible to accurately figure out what caused them. There is speculation that the impairment of the anterior chamber angle by the vitreous lysis material could be the cause of glaucoma and injury to the retina or lens resulting from an erroneous perception of distance [19, 20]. No eyes in the current study exhibited elevated intraocular pressure following Nd:YAG vitreolysis.

The Nd:YAG laser emits a high-energy pulse laser. A high concentration of photons acts on vitreous floaters, ionizes vitreous turbidity molecules, and forms plasma. In this process, the molecules will generate high-speed motion so that the temperature of the plasma center rises sharply, and the rapid expansion causes, in a very short time, tiny explosions that emit shock waves of a certain intensity. A previous study looked into the safety of Nd:YAG laser vitreolysis on rabbit eyes. The laser treatment (5 mJ x 100 pulse or 10 mJ x 50 pulse) was given to the front, middle, and back vitreous humors, and changes in the retinal layers’ vacuoles were seen [12]. The precise localization of the vitreous opacity proved challenging to ascertain with precision throughout the Nd:YAG laser vitreolysis procedure, a technique that is highly reliant on the practitioner’s expertise. As standards, the thickness of the lens or the background blurring of the retina were typically employed. Because the length of the vitreous cavity of the rabbit eye is about 5.7–6.1 mm [21], it is challenging to differentiate between the anterior, middle, and posterior segments of the vitreous when performing Nd:YAG laser vitreolysis on rabbit eyes. To avoid accidental injury to the posterior capsule of the lens and retina by the Nd:YAG laser, laser treatment was limited to the middle vitreous, with the laser’s focal point approximately 3 mm away from the lens’s posterior capsule and the retina in the current experiment. As per the manufacturer’s guidance, a maximum of 500 laser spots should be utilized during a single treatment session when employing Nd:YAG laser vitreolysis for therapeutic purposes. Occasional use of the laser machine’s maximum laser energy setting of 10 mJ/pulse on cataract patients is observed. In this experiment, the greatest number of laser spots (500) and the highest pulse power (10 mJ/pulse) were employed to validate the safety of Nd:YAG laser vitreolysis. Notably, no pathological alterations in the retinal layers were detected.

Kameel et al. [12] considered that Nd:YAG laser vitreolysis could cause retinal oxidative damage; however, vitreous AsA and TRAP were not measured in their study. Bergandi et al. [22] found that posterior capsulotomy with an Nd:YAG laser could cause oxidative stress in the aqueous and vitreous humors of humans. However, vitreous samples from patients with posterior capsular opacification before laser treatment were not collected in their study, which may have led to bias. In the current experiment, there were no significant changes in AsA and TRAP after Nd:YAG laser vitreolysis.

Although a few patients reported that their floaters got worse after receiving Nd:YAG laser vitreolysis [23], this in vivo study found that the laser vitreolysis therapy did not result in the development of any new floaters. Nguyen et al. [24] also reported that the vitreous echo density of Nd:YAG-treated eyes was 23% less than that of untreated eyes with vitreous floaters. Therefore, deteriorating symptoms may be caused by a shift in the position of floaters within the vitreous cavity or the fragmentation of large floaters into smaller ones.

The findings of this research indicate that vitreolysis with the Nd:YAG laser does not induce substantial alterations in the vitreous microenvironment or pathological changes in the retina. Nd:YAG laser vitreolysis is exceptionally well tolerated by rabbits; thus, it may serve as a viable and secure therapeutic alternative for floaters.

## Data Availability

The data supporting the fndings of this study are available within the article and for further details on requests from the corresponding author.

## Abbreviations

Nd: YAG:Neodymium:Yttrium-aluminum-garnet
TUNEL: Terminal deoxynucleotidyl transferase dUTP nick end labeling
H&E: Hematoxylin-eosin
ELISA: Enzyme-linked immunosorbent assay
VEGF: Vascular endothelial growth factor
IP: Interferon-inducible protein
MCP: Monocyte chemoattractant protein
IL: Interlenkin
AsA: Ascorbic acid
TRAP: Total reactive antioxidant potential
TBARS: Thiobarbituric acid reactive substances
VDM: Vision degrading mydesopsia
IOP: Intraocular pressure
TEAC: Trolox equivalent antioxidant capacity
RPE: Retinal pigment epithelium
DAB: 3,3′-Diaminobenzidine
ANOVA: Analysis of variance
OCT: Optical coherence tomography
